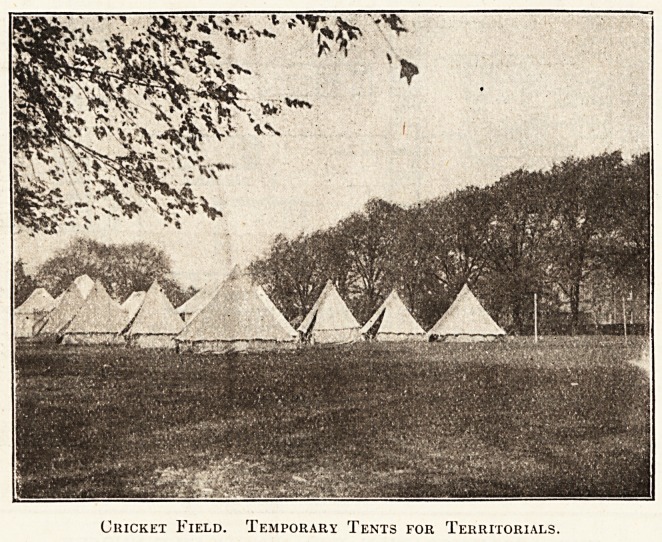# The Fifth Northern General Hospital, Leicester

**Published:** 1914-10-10

**Authors:** 


### THE FIFTH NORTHERN GENERAL HOSPITAL, LEICESTER.

At the outset instructions were given to Mr. S. Perkins Pick, F.R.I.B.A., the eminent architect of Leicester, to convert the Technical and Art Schools and two other schools for the purposes of providing the usual military unit of 500 hospital beds for non-commissioned officers and men, together with twenty beds for the accommodation of officers. These plans were actually approved by the War Office, but the serious interference with the educational organisation in the town of Leicester which must have ensued led to the relinquishment of the original scheme and the substitution for it of a new plan, which embraced the conversion of the old Leicester and Rutland County Asylum, which had remained unoccupied for several years, for the purposes of a military hospital. These latter buildings were therefore commandeered for the purpose, and Mr. S. P. Pick was instructed to prepare plans for their adaptation to the new purposes to which they would be put. The buildings stand upon a site of thirty-seven acres, which are within the borough boundary of the town of Leicester. The site consists of the highest land in the borough, being about 300 feet above Ordnance datum. The site is described as all that can reasonably be desired, and there are numerous well-grown trees, both in the grounds and in the immediate vicinity of the site. Formerly it was occupied by about 470 lunatics, the buildings being partly of two and partly of three storeys in height, whilst the administrative accommodation was contained in buildings of one-storey only. The architect has shown considerable ingenuity and care in re-arranging the old buildings so as to accommodate 358 beds in eight wards in the main block. He has provided a further 108 beds in two two-storeyed detached buildings, twenty-six beds in two floors of another building, formerly a laundry block, and twenty-eight beds in a new block, making provision capable of containing a total of 520 beds.

Of course, it was not possible to attempt to make an up-to-date hospital in connection with buildings originally planned for a totally different purpose, many of which date back as far as 1830. Exactly what it has been possible for the architect to provide will be easily realised from a study of the plans which accompany this article, and by the following brief facts.

For instance, the floor area per bed varies from 68 feet super. to 99 feet super., with an average of about 90 feet super. The height of the wards is generally about 11 feet in the clear. The conversion was rendered somewhat costly by the circumstance that the buildings had been unoccupied for seven years, and that for many previous years the Visiting Committee had spent as little as possible upon them. Further, it was necessary to pull down a large number of high walls built to enclose the airing courts and sundry other buildings, including portions of the old laundry block and the caretaker's house. With a view to economy much of the building material thus provided by the old walls has been used by the architect.

Surgical Block.
Operating Room on right of Entrance Door. Was a Chronic Block.

Administration.

The ground floor of the centre building facing north is devoted to administration. It contains the receiving room and accommodation for the officer in charge, the clerk's and registrar's offices, with accommodation for the orderly medical officers, the matron, the pay office, and the pathological laboratory, with the necessary lavatory and sanitary accommodation. The architect has been kind enough to supply us with the following details of the distribution of the accommodation he has provided by making the buildings as complete as possible for the purposes of a military hospital.

Behind the main corridor there is a telephone room, dispensary, and an orderly's room. Occupying a central position to the south-west of the official building are the two kitchens, scullery, cooks' room, bread room, two larders, knife room, and lavatory. The kitchens, being in the centre of the buildings, are well placed to serve not only all the wards, but, by two lifts, the nurses' dining room, which is immediately over, and the convalescent patients' dining room, adjoining that of the nurses. To the latter patients' dining room a separate outside staircase has been erected, which prevents patients having to traverse either the official or kitchen buildings, as well as providing an essential alternative fire-escape. The messrooms for the orderlies are situated in a one-storey building to the south-west of the kitchen, and the sergeants' dining room in a similar building placed on the north-east of the kitchen. It will thus be seen that the whole dining accommodation is fairly adjacent to the kitchen block.

### Stores.

A quartermaster's office, together with his stores, are located in a building adjacent to the main central road up to the kitchen, and also near thereto. The linen, bedding, and pack stores are separately provided for in a building to the south-west of the centre, and near to these stores are also located the dirty linen stores, the infected linen being kept in a separate enclosure from that of the ordinary soiled linen. On the first floor, over the official block, provision is made for thirteen nurses' beds, together with a large nurses' sitting room, sisters' sitting room, matron's bedroom, two bathrooms, and two w.c.s. The housemaids have a small wash-up room adjoining the nurses' dining room, and adjacent thereto a small sitting room. On the opposite side of this corridor are the two rooms for the assistant matron.

On the second floor over the official block beds are provided for thirty-one nurses and maids, with w.c. and lavatory accommodation On the second floor on the north-east and south-west of the main building is found provision for nine nurses' beds in each, and over the centre of the wings on either side are provided twenty nurses' beds, making in all a total provision of 102 beds for nurses and maids.

The Operation Room.

### Wards and Isolation Block.

To make the old buildings suitable for hospital wards the whole of the doors to the small single rooms have been entirely removed. The small windows to these rooms have been taken out, the openings increased in size, and new windows fixed throughout. This has necessarily been a large and expensive matter, but it was essential to provide more light and air than formerly existed in these buildings.

There are eight wards in the main building, each one containing about forty-four beds. The nurses' room conveniently occupies the angle room in about the centre of each ward. A linen room, ward scullery, bathroom, sink room, w.c.s, and lavatories have nearly all been refitted, and there is provided in each ward a separate nurses' w.c.

This description shortly particularises the remodelling the eight large wards in one building, No. 4 on the ground floor and No. 4 on the first floor.

In addition to these, there are two five-bed wards on the second floor, one to the south-east and another to the south-west of the main blocks, which have to be used in conjunction with the wards below. A detached two-storey modern building to the south-west provides accommodation for fifty-four surgical beds. An operation theatre, together with wash-up and anæsthetic room, all conveniently adjacent, have been adapted out of existing rooms, and rooms for *x*-ray work have also been fitted up above these rooms.

The lavatory, baths, w.c.s, and sink rooms in this block are of a better character than those in the older main building, and, generally speaking, the provision in this ward is quite good. The building needed little alteration to make it suitable for hospital purposes.

A similar detached block to the south-east of the main building provides accommodation for fifty-four beds, and it is intended to use this block for typhoid cases. The provision here is also very good, because the building is a modern erection and suitable for the purposes required. As further provision for typhoid cases, a new ward block for twenty-eight beds has been erected with old material to the north-east of the foregoing, and it has been connected up to the existing annex, which will serve the old block as well as the new block.

The administration rooms to the new block consist of linen room, bathroom, sisters' room, and ward scullery.

A two-storeyed officers' ward, providing accommodation for twenty-six beds, has been made out of the existing buildings, and a new sanitary annex has had to be erected in connection with this ward. This shortly describes the 520 hospital beds provided.

These buildings are all constructed with old bricks, the floors are laid with *in situ* cement, the roofs constructed with wood joists and boarding, the whole covered with ruberoid. They are heated by hanging steam-pipes, and the lighting is by electricity.

The outlying buildings consist of a guard-house and huts for the accommodation of sixteen sergeants and thirty-two orderlies, with necessary lavatory and other accommodation, including a canteen building. These buildings have been constructed out of the old material already referred to. The old mortuary has been made suitable for hospital use, and the following particulars relating to the sanitary and water supply may prove suggestive to those who may have to provide similar accommodation elsewhere.

Sanitation.

The sanitation of these buildings has been a source of considerable anxiety. The whole of the old main drains have been thoroughly cleaned, flushed, and disinfected, and where the main drains pass under the buildings, with one exception, they have been taken up and substituted by cast-iron pipes jointed with lead. The short lengths of drain from the mains up to the buildings have in many cases been relaid. The defective gulley traps have been removed and substituted by plain syphon earthenware patterns. Additional manholes have been provided, and ventilating pipes from all the drains have been carried up the buildings.

The system of drainage has been intercepted from the town sewer, which is separately ventilated by a tall shaft. In many cases it has been possible to leave in the old fittings, which have proved satisfactory. In other cases new closets, baths, w.c.s, slop hopper sinks, etc., have had to be provided. The whole of the sanitary system has been thoroughly overhauled, and every confidence is expressed that the building is now quite a safe one to occupy, providing reasonable care is taken to flush and disinfect the sanitary system.

Water Supply.

The water supply is obtained from the Leicester Corporation, and the water is derived mostly from the Derwent Valley Water Works in North Derbyshire, about seventy miles distant. The water is soft and of excellent quality.

Many of the water mains have had to be renewed. The remainder have had the rust bored out and the pipes thoroughly cleaned. The water standing pressure is about 70 lb., and the running water pressure about 13 lb. to the inch. It will thus be seen that for fire purposes the pressure is not very good. It has therefore been felt necessary to purchase a number of chemical fire extinguishers, which have been distributed about the buildings, particularly in those at the higher levels. There is also additional safety in the fact that the Leicester Corporation Fire Brigade are in telephonic communication with the buildings, and the fire station is within one mile of the hospital.

Care has been taken to provide a good supply of hot water throughout the establishment. The cooking plant consists partly of gas ovens and partly of steam plant, the former being leased from the Leicester Corporation. Another plant has had to be provided in order to make the cooking arrangements as complete as possible. The hospital is lighted throughout by gas. The heating is mainly by open fireplaces, with gas radiators where no fireplaces exist.

Commendable Energy.

The orders to proceed with the work were not given until August 6, and within ten days—that is, by August 17—through the zeal and strenuous efforts of all engaged on the work, over 100 beds were ready for the reception of patients, which number was increased to 470 by September 16, and the whole 520 beds were complete and ready for occupation by September 30. It was decided to contract with the public laundries of Leicester to undertake the whole of the washing of the hospital, with the exception of the infected linen, which is dealt with at the Leicester Corporation Fever Hospital. As showing the hearty co-operation of everybody in the equipment and working of this hospital we may mention that the sterilising is being done at the Royal Infirmary, the managers of which have extended their plant for the purpose, whilst the disinfecting has been undertaken by the Leicester Corporation Sanitary Department. The total cost of the new buildings and the reconstruction amounted to £10,000. To this sum must be added £5,000, being the cost of equipment. The first batch of patients was admitted on September 2, and the hospital is now working smoothly and well.

Cricket Field. Temporary Tents for Territorials.

## Figures and Tables

**Figure f1:**
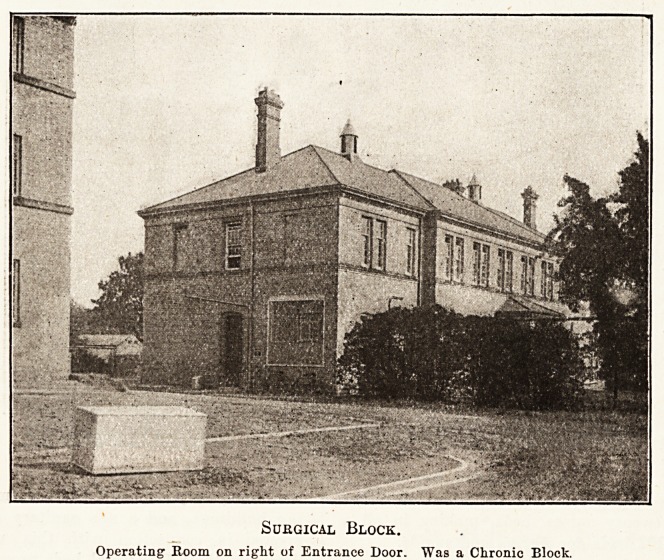


**Figure f2:**
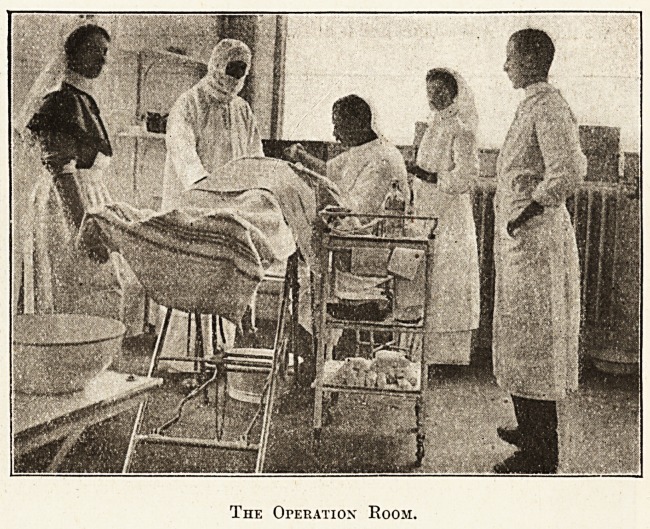


**Figure f3:**